# Supplementary figures and images for: A steady-state model of microbial acclimation to substrate limitation
Source: PLoS Comput Biol. 2020 Aug 26;16(8):e1008140. doi: 10.1371/journal.pcbi.1008140 (PMC7478835; doi:10.1371/journal.pcbi.1008140)

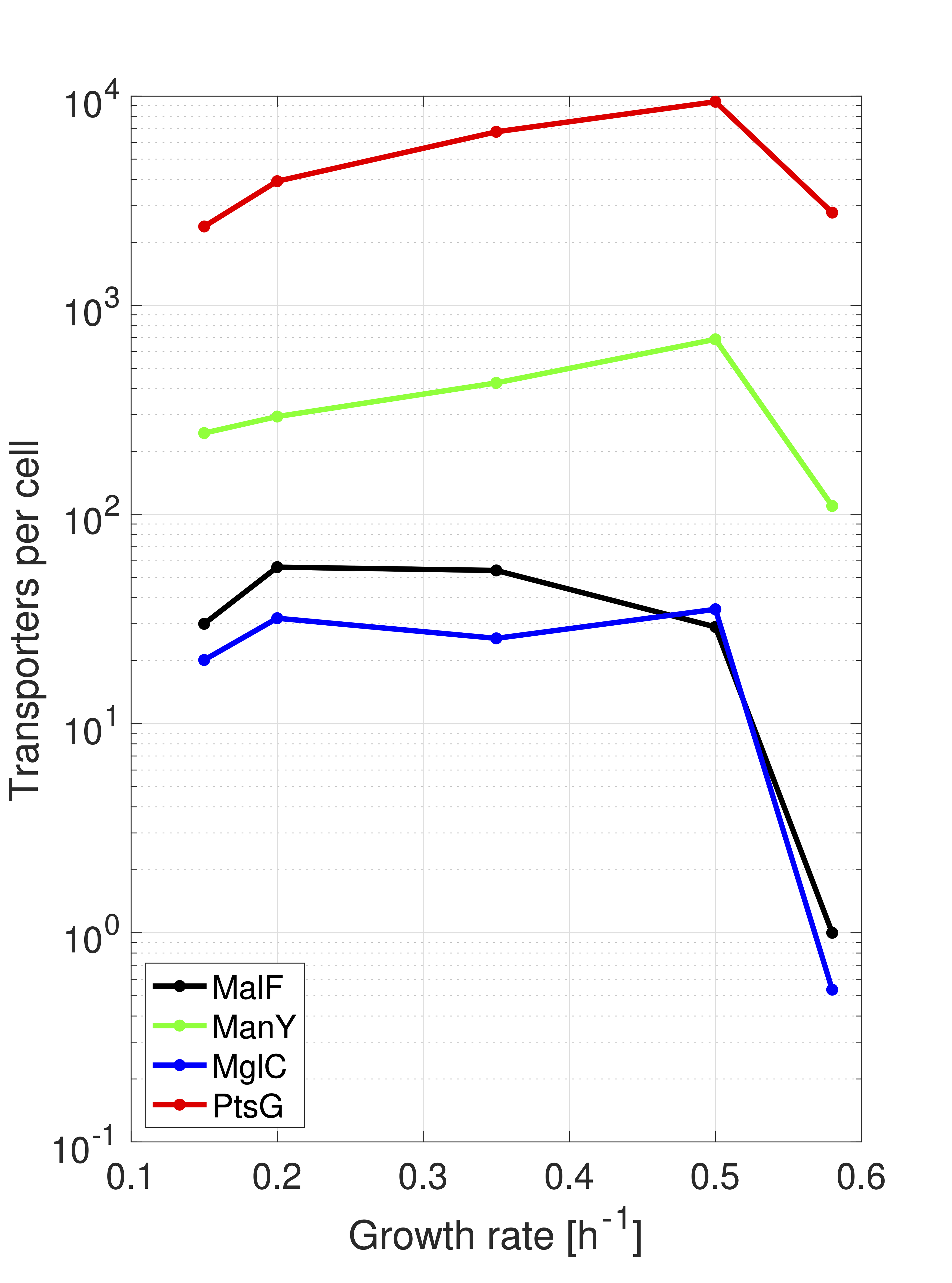

Supplement: S1 Fig — MalF—maltose permease; ManY—mannose permease; MglC—galactose permease; PtsG—glucose permease. (TIF) [file pcbi.1008140.s001.tif]
