# Supplementary material for: A steady-state model of microbial acclimation to substrate limitation
Source: PLoS Comput Biol. 2020 Aug 26;16(8):e1008140. doi: 10.1371/journal.pcbi.1008140 (PMC7478835; doi:10.1371/journal.pcbi.1008140)
Supplement: Attachment — Submitted filename: Response_to_reviewers.docx [file pcbi.1008140.s002.docx]

Dear Drs. Cannon and Alber,

We appreciate the reviewers’ thoughtful comments and feel they have improved the clarity and presentation of the manuscript. Following the suggestion of Reviewer #2, we have expanded our model to address cell size effects, and updated our model schematic (Fig. 3) to help guide the reader through this nuance. This effort was fruitful, as it allowed us to more generally state a key point in the paper relating to the transition from diffusion limitation to growth limitation, taking into account a scenario where an intermediate limiting state (surface area limitation) is encountered. Although this intermediate state was not relevant to our particular case study, the expanded model allows us to propose a mechanism to reconcile hyperbolic and piecewise-linear kinetics (a long-standing debate which, to our knowledge, has not been shown by any other mechanism). Below are our responses (indented) to each reviewer’s comments (italics) and locations of relevant changes (bold).

Sincerely,

John R. Casey

Michael J. Follows

Reviewer #1

*The authors estimated the optimal uptake rate of substrates using FBA. It is unclear how did they achieve it. Did they fix the growth rate and minimize the substrate uptake rate? It is worth explaining this procedure in the text.*

- Thank you for pointing this out, as you mention, there are a number of approaches to estimating uptake rates with FBA. Our approach was to constrain the upper bound of the biomass synthesis reaction to each experimental growth rate. We allowed unbounded uptake of all medium components, and then solved the usual FBA linear program with a subsequent L1-norm minimization of the sum of fluxes to get rid of loops that the solver often generates. This approach avoids the assumption that cells minimize uptake (which may not be the case when supplied in excess; e.g., in mixotrophic growth). We did explore a number of other approaches, including one based on a somewhat-dated study (Andersen and Meyenburg, 1980; J. Bacteriology 144(1), 114-123) wherein ATP production by oxidative phosphorylation was identified as the rate limiting growth in aerobic batch cultures of *E. coli*. Although it seemed to make sense to set a boundary constraint on ATPase and allow other relevant exchange fluxes to be unbounded, forcing ATP limitation resulted in wild variations in flux distributions (something I should have expected from FVA, but still surprising). In a brute-force approach (mostly for sanity checks), we also tried constraining the uptake rate of each substrate to a range of values, solving the more typical FBA problem for maximum biomass growth, and then identifying the closest match to the observed growth rate. This approach, while ugly, produced almost identical results to the approach we applied in this paper. We have elaborated on our FBA method to help clarify (**L211-215**), but have tried to avoid clouding the focus by restating the FBA linear program (which appears in literally thousands of articles).

*The authors claim that they used the experimental transporter numbers and equation 4 to calculate the Kcat. Is it a single point estimate? How many growth rate vs transporter number data sets did they use to calculate Kcat.*

- For the sole carbon substrates, kcat was determined from the average of three replicates of each batch culture, though we did not report error associated with the analytical variance from the proteomics dataset. However, for sulfate, phosphate, and zinc, kcat was determined across all growth conditions since they were always supplied in excess. The error associated with these substrates is somehow more meaningful (a test of the assumption that cells do not maintain ‘extra’ transporters), so we now include a statement about their variance to give the reader an idea of how stable the kcat predictions were (**L280-282**). The coefficient of variation was always less than 25% (median CV = 9%).

*In the manuscript the authors compared the model predicted transporter numbers with experimental values. It would be worth comparing the transporter number profile predicted by other models under different substrate concentrations also. This would help understand how different models fair in comparison to the experimental data.*

- Fig. 1 is intended to serve this purpose, even if in a cartoonish manner. Part of the motivation for this model was to enable the direct quantitation of the necessary parameters to compute uptake rates for an arbitrary substrate and microbe, since the previous attempts (articles advocating models A and B) made parameter estimation a bit of a guessing game (making this cartoon not qualitatively different from a fitting exercise or an otherwise arbitrary parameterization of either model A or B). As Fig. 1 shows, neither model (A or B) will fit the observations, regardless of the parameterization.

*Authors assume that under low concentrations the diffusion limits the uptake rate. However, when the external environment is well mixed the rate limiting step will be the encounter rate. Will the proposed model for acclimation hold under this condition?*

- The model accounts for this effect with the Sherwood number (Sh) parameter which appears in the diffusive limit to the half saturation concentration. Sh is a dimensionless quantity which is proportional to the ratio of the advective shear forces to the viscous forces within a volume defined by a characteristic length scale of the boundary layer. This parameter also influences the critical substrate concentration S*, such that increasing the advective shear (through stirring, for example) will decrease S* and thus alleviate diffusion limitation. For simplicity, we have chosen to ignore variations in Sh although this parameter may be of interest to ecological modelers when considering the effect of turbulence on nutrient limitation. We have added a similar statement in the revised manuscript (**L122-123**).

*The authors compare their prediction with the chemostat data. How did the authors confirm that the highest dilution rate corresponds to the growth limit? In the chemostat it will be very difficult to reach the growth limit as it would lead to wash out.*
 - The maximum growth rate for which we compared glucose-limited chemostat data to was derived from the glucose-replete batch culture (0.60 h^-1^), not from the highest dilution rate chemostat (0.50h^-1^). These are quite close, but with enough wiggle room to avoid washout as you mention. Since the critical transition concentration S* is derived from the glucose-replete batch condition alone, the model provides an independent assessment of the steady-state glucose concentration in the highest-possible dilution rate chemostat (0.6 h^-1^), if one were practically possible. The similarity between S* and the observed concentration is, hopefully, an encouraging result.

Reviewer #2

*The authors note in line 154 that for ambient substrate concentrations below the nutrient-limited concentration (which they term the diffusion limited regime), that there is a dependence on the cell radius (i.e., size) and that volumetric changes at different glucose-limited dilution rates. The authors should describe what modifications to the model would need to be made in order to account for the change seen in E. coli and to species with greater observed changes in cell size.*

- Thank you, we have expanded our discussion and mathematical description of cell size effects in the Model section (**L188-199**) and have introduced two additional equations which formalize the transition to surface area limitation (**equations 16 and 17**), and an accompanying equation to account for the additional limiting state (**equation 18**). To accommodate these changes and to help the reader navigate what is a somewhat nuanced aspect of the model, we have also added a ‘small cell’ scenario to our model schematic (**Fig. 3 panels B and D**). This line of inquiry led us to a possible mechanism for reconciling hyperbolic and piecewise-linear models (thank you!), which we now elaborate on a bit in the Discussion section (**L352-354**). Although it will not appear in this article, we have incorporated this model into a larger optimization problem which takes into account a variety of other cell processes that make an optimal cell size prediction more meaningful than accounting for nutrient uptake alone, so more to come.

*In the Model Validation section (beginning on line 229), the authors describe some of the results from the model and comparisons with literature values, most of which are the same order of magnitude. The authors adequately discuss differences with zinc, which had the greatest difference, but should also discuss the differences observed with acetate.*

- It seems that this strain expresses an additional transporter that has some specificity for acetate that may be complicating the picture (biphasic kinetics). We have added a brief explanation along these lines (**L265-269**).

Reviewer #3

*It would be nice to have data points for glucose concentrations between 10 uM and 30 mM in Figs 1 and 4. However, I understand that if the authors are relying on existing data, adding these data points may not be feasible.*

- It certainly would! Populating the disparity between glucose replete (33 mM) and glucose limited (about 8 microM in the dataset we analyzed) will be a welcome addition, and one that we will be tracking as quantitative proteomics data become more commonplace.

*Please consider explicitly mentioning what parameter is being fitted for the model in Fig 4.*

- Figure 4 shows a direct comparison between predicted and experimentally determined half saturation concentrations. The solid line is not a fit but rather the 1:1 line which was intended only as a visual guide.

*If I’m interpreting Eq (14) and Fig 5 correctly, the model assumes that at high concentrations of glucose the growth rate does not change. In our empirical observations with E. coli growth (coincidentally an MG1655 strain) at different glucose concentrations, the growth rate remains fairly steady at low and intermediate levels of glucose, but drops at very high concentrations (e.g. a significant drop at 20 mM of glucose). Since a change in the growth rate means a change in the biosynthetic rate (Fig 2), I am curious if such a drop is either predicted from the current model or maybe alternatively (if it is caused by another mechanism) partially explains the lower transporter numbers at high glucose concentrations.*

- Thank you for alerting us to this phenomenon, which we were previously unaware of. Our model currently does not consider any substrate concentration dependence of V_max_^G^, but the growth rate of the glucose replete batch culture during exponential phase was 0.60 h^-1^ in the Schmidt et al., 2015 study was at least comparable to growth at somewhat lower concentrations (e.g., 0.57 h^-1^ at 16 mM; Paalme et al., 1995, Antonie von Leeuwenhoek 71, 217-230). This is by no means an exhaustive literature search, but more in line with your experience, these growth rates are higher than those reported for “high glucose” (0.40 h^-1^ at 250 mM; Marisch *et al*., 2013, PLOS One 8(8), e70516). Perhaps the transition is more difficult to spot when comparing across different studies, and we will certainly be interested to apply our model to future proteomics datasets which fill in the gap. Also, I felt it was a bit too anecdotal to include in the text, but we often see a ‘hump’ in transporter gene expression levels (moving from nutrient-starvation to nutrient limitation to nutrient replete; e.g., Krumhardt *et al*., 2013 Environmental Microbiology 15, 2114-2128 and accompanying paper Reistetter *et al*., 2013 Environmental Microbiology 15, 2129-2143) in batch cultures of marine organisms grown on a variety of nutrient sources, suggesting this behavior is general. It is quite a stretch to make the jump from transcripts to proteins and often these observations were not strictly steady-state, so we avoided the comparison but they are nonetheless consistent with our model.

*Possibly relevant to the previous comment, when comparing with experimental data, it would be helpful (although not necessary) to estimate up to what concentration the assumption of “growth under a single limiting resource” is valid.*

- This is central to the paper, and we have clarified the meaning of S*, which is a concentration that marks the (steady-state) transition between growth limitation and diffusion limitation. This point was unclear in the original submission, and we have corrected its formal definition (**equation 14**) and expanded on its relationship with surface area limitation (**equations 16-18; L188-199; L352-354**).
